# Interactive Design Psychology and Artificial Intelligence-Based Innovative Exploration of Anglo-American Traumatic Narrative Literature

**DOI:** 10.3389/fpsyg.2021.755039

**Published:** 2022-02-10

**Authors:** Xia Hou, Noritah Omar, Jue Wang

**Affiliations:** ^1^College of Foreign Languages, Zhoukou Normal University, Zhoukou, China; ^2^Department of English, Faculty of Modern Languages and Communication, Universiti Putra Malaysia, Serdang, Malaysia; ^3^School of International Studies, Hunan Institute of Technology, Hengyang, China

**Keywords:** artificial intelligence, interactive design, psychology, Anglo-American literature, trauma narrative

## Abstract

The advent of the intelligence age has injected new elements into the development of literature. The synergic modification of Anglo-American (AAL) traumatic narrative (TN) literature by artificial intelligence (AI) technology and interactive design (ID) psychology will produce new possibilities in literary creation. First, by studying natural language processing (NLP) technology, this study proposes a modification language model (LM) based on the double-layered recurrent neural network (RNN) algorithm and constructs an intelligent language modification system based on the improved LM model. The results show that the performance of the proposed model is excellent; only about 30% of the respondents like AAL literature; the lack of common cultural background, appreciation difficulties, and language barriers have become the main reasons for the decline of reading willingness of AAL literature. Finally, AI technology and ID psychology are used to modify a famous TN work respectively and synergically, and the modified work is appreciated by respondents to collect their comments. The results corroborate that 62% of the respondents like original articles, but their likability scores have decreased for individually modified work by AI or ID psychology. In comparison, under the synergic modification efforts of AI and ID psychology, the popularity of the modified work has increased slightly, with 65% of the respondents showing a likability to read. Therefore, it is concluded that literary modification by single ID psychology or AI technology will reduce the reading threshold by trading off the literary value of the original work. The core of literary creation depends on human intelligence, and AI might still not be able to generate high-standard literary works independently because human minds and thoughts cannot be controlled and predicted by machines. The research results provide new ideas and improvement directions for the field of AI-assisted writing.

## Introduction

Traumatic narrative (TN) originates from the Anglo-American (AAL) literary world in the 20th century which is shaped by many traumatic events, such as World Wars, military conflicts, revolutions, and genocides. These traumatic events not only have caused millions of fatalities among civilians and soldiers but also have brought physical, as well as psychological, pain to numerous families. At that time, some AAL writers who have witnessed the suffering of the people write down these historical tragedies through literary creation, which then gradually has evolved into AAL TN literature (Jill, 2019; Alpert et al., 2020). Linguistically, the term “trauma” is originally used to describe physical injury caused by external forces (Pathis and Place, 2018), while “trauma” in the context of war can narrate the psychological suffering behind physical pain. Freud (Maureen, 2018) is the first person to put forward the “trauma theory.” It is believed that the best way to alleviate or treat “trauma” is through conversation or transcription (Maxence, 2019; Ellison, 2021). Therefore, after a series of major trauma events, TN literature naturally comes into being to record the process of human development, remind them of the cruelty of war, and help them learn from history to choose the right path toward civilization (Liu, 2019). This article studies TN in the 21st century and provides some references for the development of this far-reaching type of literary works.

With the advent of the 21st century, artificial intelligence (AI) technology has been seeing broader applications in various fields, including the field of literary creation, thereby replacing heavy and repetitive manual labor (Smedslund, 2021). At present, AI-assisted writing is mainly used in news scripts with strong formality and regularity (Yu and Lin, 2020). Still, the inspiration and unique writing skill styles of writers are something AI cannot mimic, whereas they are the key to literature creation; thus, AI technology still cannot take the place of the complex human literary creation (Mullholland, 2020). Thereupon, through the analysis of AI technology, this study mainly discusses the auxiliary role of AI technology in AAL literary creation. Meanwhile, interactive design (ID) psychology is introduced to study the synergistic effect of ID psychology and AI on AAL literary creation. TN literature is one of the most influential literary types in the 20th century, as well as the most widely developed literary type in AAL literature (Newman, 2021).

It is of practical significance to study the AAL TN in the era of AI. First, this study introduces the theoretical basis of AI and ID psychology; second, combined with the Questionnaire Survey (QS) method, the influence of AI and IS psychology is studied on TN literature; finally, the results of the QS are analyzed. This study concludes that the synergic literary modification of AI technology and ID psychology can provide people with a new reading experience of AAL traumatic narrative writing. For example, it can reduce the reading threshold by simplifying the content and structure, but the improvement effect on the reading experience is not obvious. This shows that AI is more suitable for assisting writing than replacing human creation. The research content provides an improvement direction for the development of AI writing and the substitutability of human labor in some fields in the era of AI. Innovatively, this study combines AI technology with psychology for the first time and studies the TN of AAL literature under the combination of state-of-art technology and psychology. The research results can be applied to AI writing, which provides a reference for the combination of AI-assisted writing and psychological theory.

### Related Work

This section is divided into two parts, namely, the current trend of digital reading and the research status of the synergy of AI technology in ID psychology.

With the development of Mobile Internet (MI) technology and the widespread intelligent terminals, digital reading has gradually become a popular reading style. Many researchers have studied the digital reading status of different groups. For example, Banni et al. (2019) analyzed the demand and usage of digital cultural resources of migrant workers based on QS and interviews, revealing their major digital readings as current political news, e-books, music, movies, entertainment, and sports (Banni et al., 2019). Nazari et al. (2021) investigated the current situation of digital reading of teenagers and uncovered that the overall extracurricular reading time of students was decreasing, and the time spent on digital reading was increasing remarkably; the most favorite online activities of students were entertainment; reading habits of students had been reshaped by the current MI technology. The reading methods of people had been gradually changed into digital reading, and word-by-word reading had been shifted into hypertext-based skimming (Nazari et al., 2021).

Norqulovna et al. (2021) studied the contents related to ID psychology, sociology, and technical science and explored human psychology and machine capability (Norqulovna et al., 2021). In terms of the research on AI, as early as the 1950s, some researchers have predicted that AI would gradually infiltrate human production and life in the next 50 years, resulting in different opinions and factions. The researchers have described these factions and expounded on the era of AI. Mu (2021) discussed the impact of the continuous development of Science and Technology (S&T) on human society; applied some AI algorithms to the real environment; studied AI from the perspectives of philosophy, sociology, psychology, and neurology; and put forward a new perspective on the trend of “singularity.” It also showed the impact of some AI-inspired S&T phenomena worldwide (Mu, 2021).

To sum up, according to the research status both in China and outside, there are various literary works on AI in informatics and philosophy, whereas little research has been conducted on the combination of AI and ID. It is believed that with the further maturity of S&T, AI will inevitably intervene in ID. This study attempts to make research and speculation within a limited scope because the technological revolution is a dynamic process and will not stay for long in the current thinking range of people.

### Research Methods

#### Artificial Intelligence Technology

The world today has been intellectualized like never before. Especially, as a new intermediary, AI has penetrated all aspects of life and work, thereby improving employee feedback and work performance (Qian et al., 2018).

Explicitly, AI can be applied in literature creation and contribute to literary development. Literature evolution is a historical process, and literary works record and showcase history *via* an artistic form (Antonihgni et al., 2020), which, in turn, will be influenced by the historical backgrounds and technological development of their time. Originally, the TN literature is composed under the tragic war-wreckage backgrounds of the 20th century, while the modern TN literature creation has been flourished in the age of peace and has been much diversified by including various themes apart from war (Larysa, 2020).

Meanwhile, literature creation is vigorously shaped by the times and technological innovations (Aldo, 2020). In particular, literature creations and transcriptions have undergone several stages: from ancient word-of-mouth, traditional handwriting, and modern-times printed works to contemporary electronic network literature and the latest AI-assisted writing; AI, as the ultimate digital technology, will surely innovate literary creation styles. For example, intelligent literature has begun to take shapes, and AI robots such as Xiaobing and the Nine Songs provide a glimpse of the foreseeable new literature pattern despite their widely criticized literary values (Geoff et al., 2019).

Accordingly, this study makes an effort to modify a piece of AAL TN literature work by AI technology and collects the evaluations and opinions of readers on the AI-modified work through a QS design.

#### Interactive Design Psychology

The development of TN literature benefits from the deepening research on modern psychology, especially, trauma psychology. Particularly, ID psychology, as one of the emerging and most intensively discussed subfields of psychology (Larysa et al., 2018), can empower TN literature creation a new vision.

Metaphorically, trauma psychology is the internal engine of TN, and ID psychology is the wheel that carries TN onward. ID psychology can enhance interpersonal communication, facilitate the understanding and usage of users, and, most importantly, get them enchanted with the target. There are many theories of ID psychology, including the 7 ± 2 rule, Hick’s law, Von Restorf effect, and Occam’s razor principle (Deng L. T., 2021); according to these scientific bases, this study modifies a piece of AAL TN literature work, and the QS is used to collect post-reading evaluations and opinions of readers.

##### The 7 ± 2 Rule

**FIGURE 1:**
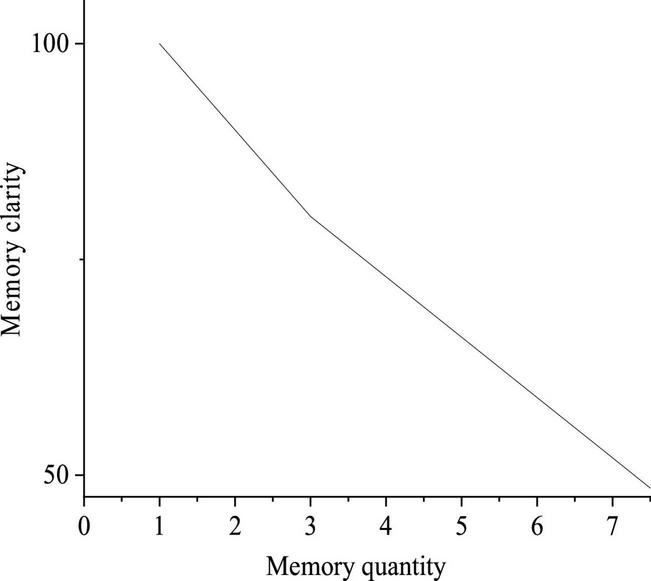
Schematic diagram of the relationship between memory clarity and memory volume.

The rule argues that the short-term memory of the human brain is approximately seven information objects, most, namely, between 5 and 9; yet, the law can explain some practical problems: the less the information is, the higher the memory clarity is, whereas the more the information is, the worse the memory clarity is (Huang et al., 2021). Figure 1 outlines the approximate relationship between memory volume and clarity.

Figure 1 displays that the memory clarity within three information objects is very high. Then, with the increase of the number of information objects, the clarity declines sharply until the memory volume equals four information objects. From then on, as the information objects continue to increment, the decline rate is less steep, but the clarity has fallen to less than 80%. Furthermore, when the memory volume reaches seven information objects, the memory clarity falls almost 50%, which is theoretically judged as easily distractable fuzzy memory rather than a clear memory.

##### Hick’s Law

**FIGURE 2:**
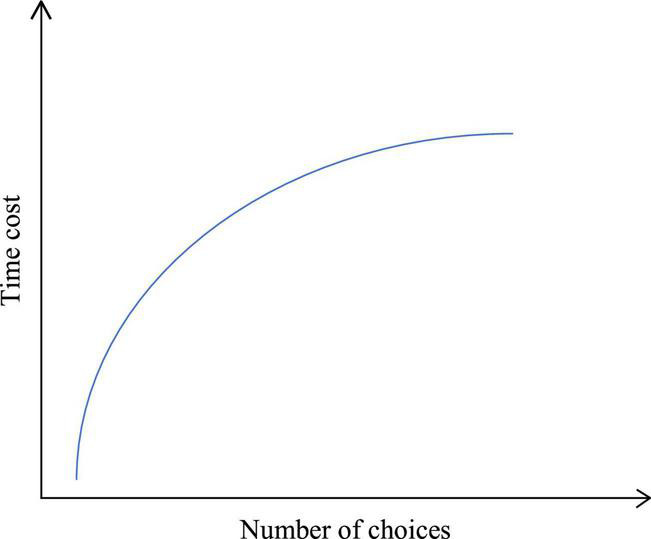
Schematic diagram of the relationship between the time cost and the number of choices.

Hick’s law is a psychophysical theorem of the relationship between the number of choices and the time cost, which is commonly described that the more choices there are, the longer time is to make a decision (Kvålseth, 2021). The approximate relationship between the time cost and the number of choices is presented in Figure 2.

Figure 2 suggests that the relationship between the time cost and the number of choices is a monotonous non-proportional one. Overall, fewer choices can ensure a reduced time cost and, thus, facilitate utilization.

##### The Von Restorff Effect

**FIGURE 3:**
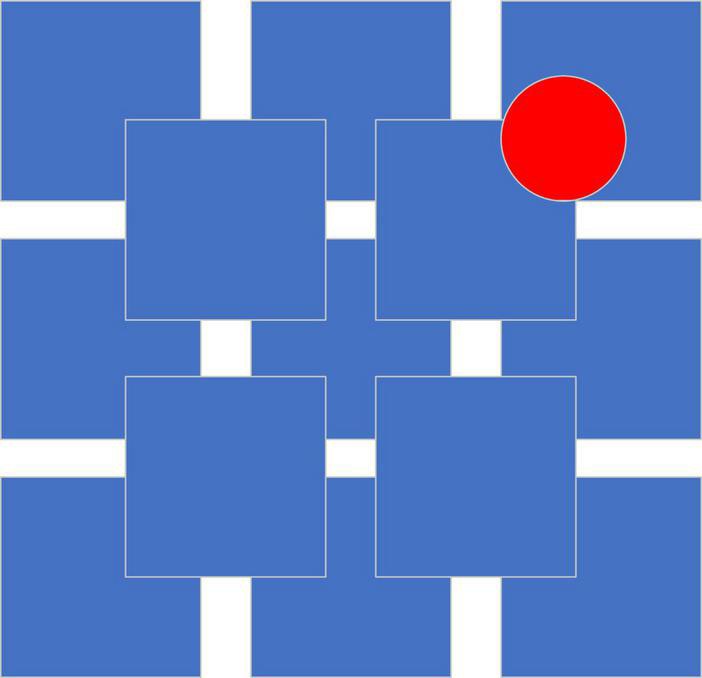
Example of the Von Restorf effect.

The Von Restorff effect, also known as the isolation effect, proclaims that compared with similar items, only special or unique items are more likely to be remembered. In other words, the more an item violates the convention, the easier it is to be remembered (Qian and Goh, 2018). Figure 3 exemplifies the Von Restorff effect.

Obviously, the red circle can attract the attention of people more and, thus, can be remembered with more information, such as location and characteristics.

##### The Principle of Occam’s Razor

**FIGURE 4:**
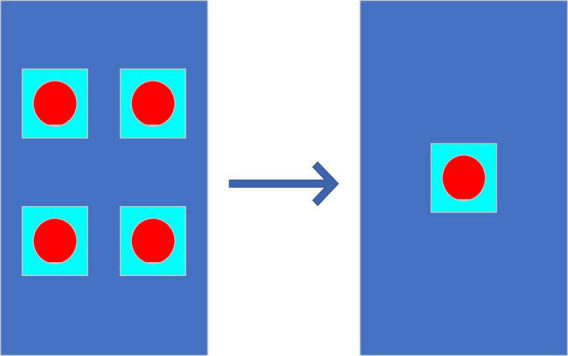
Schematic diagram of the principle of Occam’s razor.

Literally, it is to shave off the excessiveness with a razor to achieve an optimized effect with as few choices as possible; that is, there is no need for entity multiplication unless necessary, namely, the simplest effective principle (Rehfeldt, 2019). The principle is shown in Figure 4.

#### Artificial Intelligence-Related Technologies

Deep learning (DL) has been proved to be a successful experimental machine learning (ML) technology and is mostly based on neural networks (NN). In contrast, natural language processing (NLP) helps machines process and understand such information as sound and written texts in human language through relevant programs and algorithms. Thanks to the continuous technological advancement, NLP is seeing more extensive applications, with some cross-form combinatorial scenarios. For example, NLP can be used for picture description tasks, auxiliary teaching, reading pictures, and writing articles. This section mainly studies some applications of NLP technologies.

TextRank is a graph-based text summarization (TS) algorithm or rather, the improvement of the PageRank technique. The basic idea is to divide the text into several words or sentence units, then create a graph ranking model, sort the important text components by the voting mechanism, and complete the keyphrase extraction (KE) only through the information of a document itself. The expression of TextRank reads:


(1) $$\mathrm{WS}\left(\mathrm{Zi}\right)=\left(1-\text{d}\right)+{\text{d}}^{*}\sum \mathrm{ZiIn}\left(\mathrm{Zj}\right)\frac{{\text{W}}_{\text{ji}}}{\sum_{\text{Zk}\varepsilon \text{out}\left(Z_{j}\right)}W_{\mathrm{jk}}}\mathrm{WS}\left(\mathrm{Zj}\right)$$

where *d* is the damping coefficient, usually set to 0.8; Zi represents any vertex in the graph; In(Zi) denotes all vertices pointing to vertex Vi; Out(Zi) indicates the set of all vertices connected by vertex Zj; Wij stands for the connection weight of vertices Zi and Zj; WZ(Zi) means the final ranking weight of vertex Zi.

**FIGURE 5:**
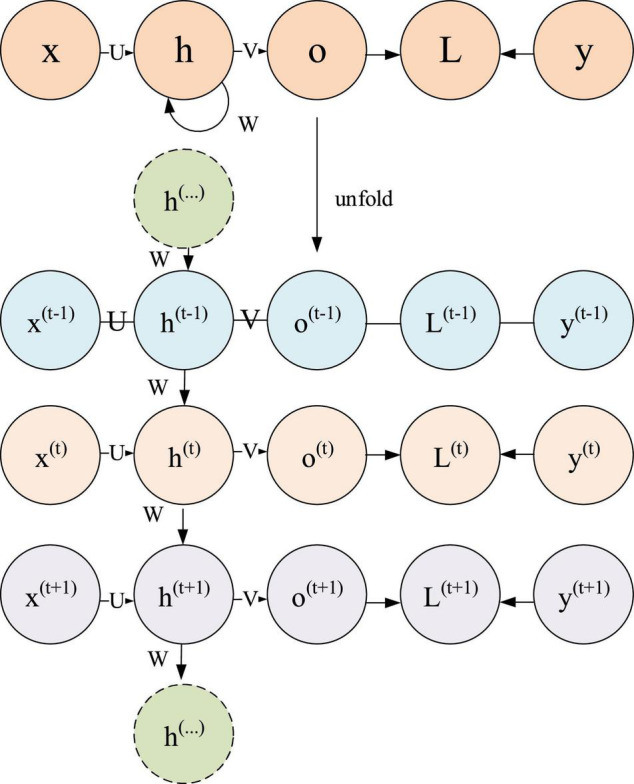
Recurrent neural network model.

The automatic text generation (ATG) or generation of a long sentence, paragraph, or article using a keyword often employs the recurrent neural network (RNN) structure because of its excellent sequence-data solving ability to automatically fill text sequence data. Figure 5 outlines the RNN model structure.

In Figure 5, the RNN model is divided into initial state and expanded state according to time series; *X*^(t)^ represents the input of the sample when the sequence number is at time *t*; *h*^(t)^ is the hidden state of the sequence number at time *t*; *o*^(t)^ denotes the output of the model at the time of sequence number *t*; *L*^(t)^ signifies the loss function of the sequence number model at time *t*; *y*^(t)^ is the actual output of the sample sequence of the sequence number at time *t*; *U*, *W*, and *V* mean matrices, which belong to linear relation functions in the model, are shared in the whole RNN, and realize the “cyclic feedback” of RNN model.

The ATG is to automatically generate a piece of text using the machine according to keywords. According to its input patterns, ATG includes text-to-text, image-to-text, and data-to-text generations. ML includes supervised learning, unsupervised learning, and reinforcement learning (RL). ML has the advantages of precision, automation, speed, customization, and scale. General ML steps cover data preparation, data preprocessing (DPP), feature extraction (FE), training model, evaluation model, and application training.

Deep learning is based on a network, which consists of many basic network structures. Overall, these network structures can be divided into three different network models: multilayer perceptron (MLP), convolutional neural networks (CNN), and RNN. As DL technology develops rapidly, various open-sourced DL frameworks have been proposed. The commonly used DL tools include TensorFlow, CNTK, Caffe, and Keras, of which TensorFlow is so far most prevailing. In particular, TensorFlow is an open-source DL framework launched by Google and contains almost all NN-related layers and auxiliary functions, which can greatly reduce the workload; in simple words, users do not even need to understand the optimization algorithm and distributed concept to complete the construction and training of deep neural network (DNN). Meanwhile, some issues have been raised from ML; for example, in the NN, a model with too many layers will cause gradient disappearance and gradient explosion.

#### Artificial Intelligence Technology and Psychology

Artificial intelligence-assisted writing originates in the United States. Western media has been using AI technology for news writing with a much higher technological maturity than their Chinese counterparts (Xu et al., 2020; Popović et al., 2021). American scholars have demonstrated in detail the technical support of AI-assisted writing and the impact of AI-assisted writing on media transformation and state that algorithms and computer programs are the two basic technical supports of AI-assisted writing. Concretely, computer programs can retrieve useful information from huge information islands, whereas algorithms can translate the coded information into the natural language (NL) format (Thameur et al., 2021). It has been argued that “AI writer” is programmed to convert data into texts based on algorithm technology (Deng Y., 2021). With the advent of the algorithm era, research on AI-assisted writing is proliferating. Some scholars believe that although AI-assisted writing has brought great convenience, it is imperative to supervise the algorithm process, specify the responsibility issues, and ensure its transparency (An, 2021). Some authorities contend that AI-assisted writing can liberate humans from heavy and mechanical writing tasks to spend more time on the literary conception that quests for peculiar human intelligence (Liu and Varatharajan, 2021). American scholars have found that AI-written news might be boring but can objectively present facts through comparative analysis with journalist–written news (Cai, 2020). AI-assisted writing has not seen its debut in China until 2015 when Tencent News applies AI technology to write news. Since then, the research on AI-assisted writing has seen a long-term and steady progress thanks to the mass Chinese media workers, as well as the Journalism and Communication majors in higher institutions (Wu et al., 2021). Currently, AI-assisted writing in China is mainly focused on news writing (Lu, 2019). To sum up, most of the existing international and domestic research on AI-assisted writing focuses on the influence and technical aspects of news writing. There is still less research on the application status, future development trend, and application of AI-assisted writing in other types of writing. This study explores the application of AI technology in AAL TN to fill the research gap.

Noticeably, AI shares commonness with art design (Yan et al., 2020). It is believed that exploring the application of AI to ID can provide more theoretical references for intelligent ID (Liang, 2019). Accordingly, this study combines cutting-edge AI technology and ID psychology to explore their possible combination patterns to make the most of the two techniques and achieve a stronger fusion effect.

**FIGURE 6:**
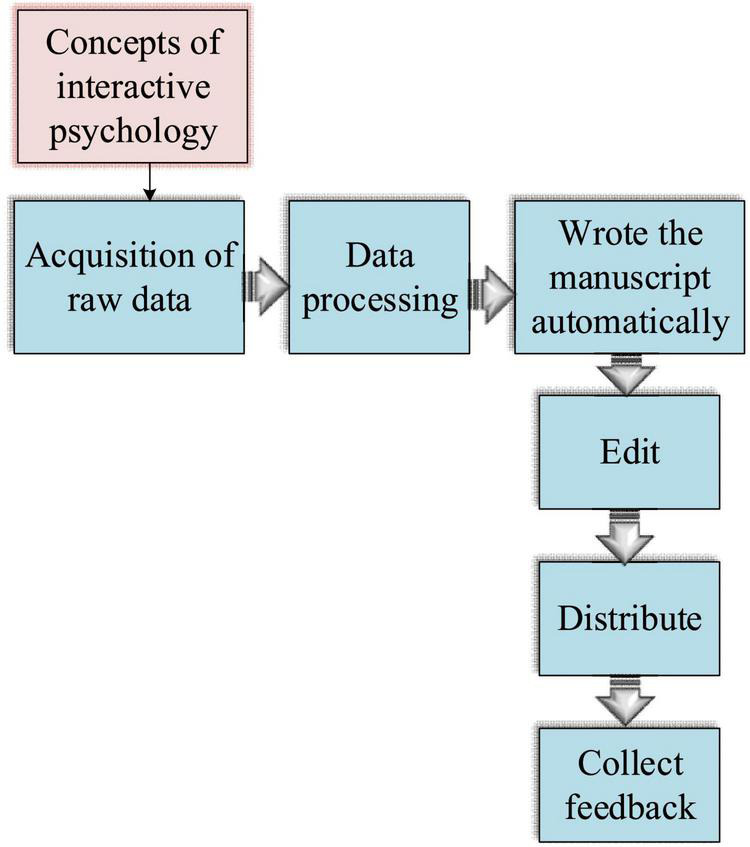
Artificial intelligence writing process based on ID psychology.

Traditional AI-assisted writing research focuses on the theoretical level and news writing only. Under the background of interdisciplinary research, exploration of the combination of AI technology and psychology is still insufficient. Although there are sporadic psychology-oriented AI studies, few have probed into the combination of psychology and AI-assisted writing. Therefore, this study combines ID psychology and AI technology to study the role of their interaction in the creation of AAL TN literature. Besides, AI technology (Chen and Chen, 2021) can greatly facilitate the life and work experiences of people, while ID psychology can bring people a more comfortable and pleasant experience (Crum, 2020). Thus, the combination of the two might produce a more promising positive effect. For example, AI technology can be used to predict the purchase behavior of customers (Verdi et al., 2020); under the ID psychology-optimized commodity display scheme, the purchasing hesitation and uncertainties of customers can be ameliorated, hence helping customers save time while profiting business more. The combination of AI technology and ID psychology asks for the support of various algorithm technologies, such as natural language generation (NLG), automatic document summarization, KE in DL, and corpus development. In particular, this study takes the fragments in *The Sun Also Rises* as the main experimental object for KE. Specifically, the AI writing depends on huge amounts of data and information collection, establishes the AI learning database, inputs the relevant concepts of ID psychology into the database, and learns specific data using the DL algorithm. According to the set template, specific writing is carried out for different manuscript types. The writing process is represented in Figure 6.

Figure 6 divides the ID psychology-based AI writing into six steps: data collection, data processing, automatic manuscript writing, editing, distribution, and feedback collection. First, concepts related to ID psychology are added to the collected data to study the TN of AAL literature (Pooja et al., 2018); then, the AI technology is combined with ID psychology (Harré, 2021); the combination technique is used to modify one piece of TN literature work; finally, a QS is specifically designed to collect post-reading evaluations and opinions of respondents on the AI-modified work.

### Construction of Machine Writing Model

The RNN model has two serious defects in text processing: first, the conventional RNN generates more weight data during text processing, which is not conducive to the model to learn more data features; second, the conventional RNN cannot solve the problem of long-time dependence of sequences. To realize the intelligent writing of machines, it is desirable for the NN structure to have the function of long-time memory of input data to be learned by machines. Therefore, this study proposes to build a double-layered RNN machine writing language model (LM) to solve the problem of too much weight data in the NN structure. At the same time, the long-short-term memory (LSTM) model with long-term memory function is used to replace RNN to solve the long-term dependence of sequence generation, and the loss of the model during training is reduced by increasing the depth of LSTM.

**FIGURE 7:**
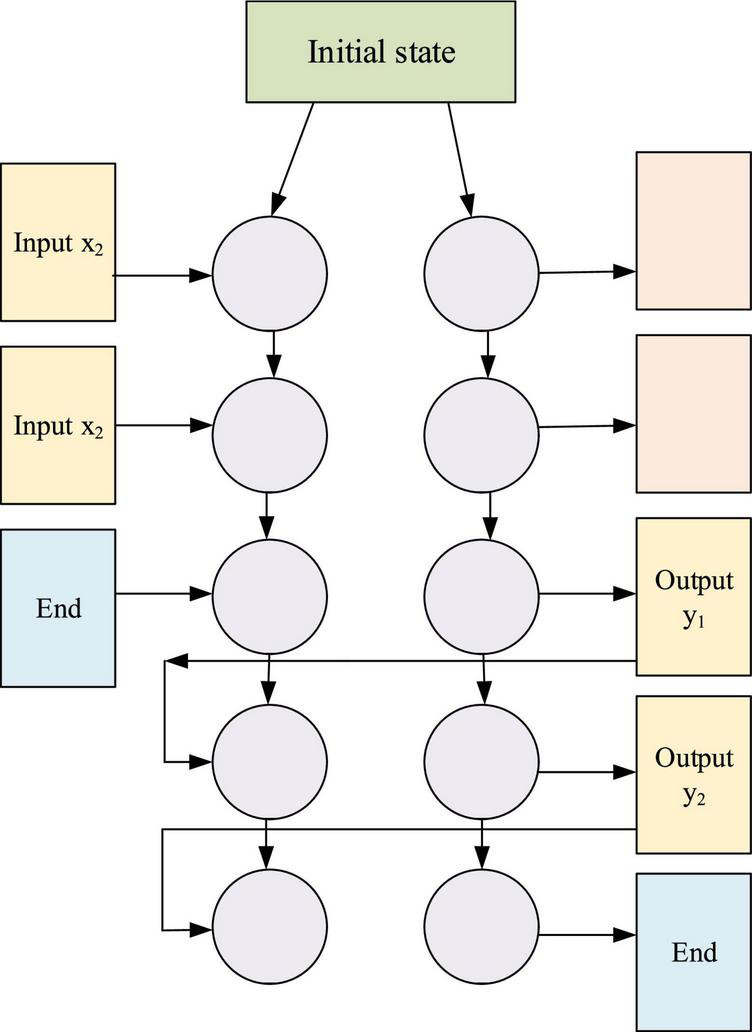
Double-layered RNN model.

The RNN algorithm model has a good ability to deal with sequence problems, so many NLP tasks can be completed by the RNN model. Especially, to solve the problem of long-time dependence of RNN, a variant of RNN, LSTM, is used in this study. LSTM is an improvement of RNN; it has an excellent performance in solving the problem of long-term dependence. LSTM is to replace ordinary neurons in RNN with LSTM units that can store a small amount of memory. Similar to RNN, these LSTM units are connected to remember the state of error in each time step; its implementation principle is that this internal state has a self-connection with a fixed weight of 1 and a linear activation function (AF). In the back-propagation stage of training, the constant weight of 1 can carry errors in many time steps without gradient disappearance. In practical application, in addition to a linear function, it also has a nonlinear AF sigmoid, and each LSTM contains two units called “Gates,” namely, input gate and output gate. One is responsible for learning to scale the incoming data, and the other is responsible for learning how to scale the value to be output. To make the LM have a better effect, this study uses double-layered RNN to construct the LM; the double-layered RNN has fewer weight data, which is conducive to the model to learn more features. Moreover, the implementation of the proposed model is based on LSTM, and it improves the effect of the model by increasing the depth of LSTM. The double-layered RNN model is expounded in Figure 7.

Figure 7 illustrates that when processing sequence data, the NN will store the output of the previous iteration, and its connection is expressed by the following equation:


(2) y=σ(Ax)

where *A*, *x*, and σ are the weight, the input, and AF, respectively; the execution of AF will return to the output layer *y*_1_.

If there are sequence inputs *x*_1_,*x*_2_,*x*_3_,…, the connection equation is modified to take the previous input into account, as shown in the following equation:


(3) $$y_{t}=\sigma \left({\text{By}}_{t-1}+{\mathrm{Ax}}_{t}\right)$$

where *B* is the weight.

The next input is obtained based on recursive iteration. The probability distribution output is obtained through the softmax function, as calculated by the following equation:


(4) $$s_{t}=\text{softmax}\left({\mathrm{Cy}}_{t}\right)$$

where *C* is the weight; *s*_*t*_ is the output at time *t*; once, there are the outputs {*s*_1_,*s*_2_,*s*_3_,…} of all sequences, and all sequence output results are fed back to the model as sequences to generate multiple output results; then, the last output result is taken as the final prediction result.

The creation of the model is realized through the proposed improved RNN. The design process of the LM includes importing relevant programming libraries, creating calculation diagrams, and setting relevant parameters; defining model folders, selecting data sets, and cleaning data; creating vocabulary, converting vocabulary into data, defining LSTM model parameters, iterative training, and outputting results. Of these, LSTM is the core of the LM. The LSTM model is designed by increasing the depth of the proposed RNN, and the number of LSTM layers in the model is set to three.

This study optimizes the model from two aspects: learning rate (LR) and input data, thus improving the application effect of the LM. LR plays an important role in supervised learning and DL; it determines whether and when the objective function (OF) can converge to the local minimum. Meanwhile, the LR can be either fixed or dynamic, in which the fixed LR is not suitable for ML because the machine needs to improve the accuracy through continuous learning. Therefore, in the process of model training, the LR is generally changed dynamically according to the number of training rounds. Here, the designed machine writing model also adopts the dynamic LR; the appropriate LR can make the OF converge to the local minimum in a specific time, reduce the gap between the prediction results and the objective results, improve the accuracy, and also help to optimize the performance of the model. According to large numbers of experimental results and previous experiences, this study dynamically adjusts the LR of the machine by slowing down the index. The expression is exhibited in the following equation:


(5) $$\alpha =a^{\mathrm{epoch}\_\mathrm{num}}$$

where *a* is a constant, 0 < *a* < 1; epoch_num represents the number of cycles; α denotes the learning rate.

### Intelligent Writing Modification System

**FIGURE 8:**
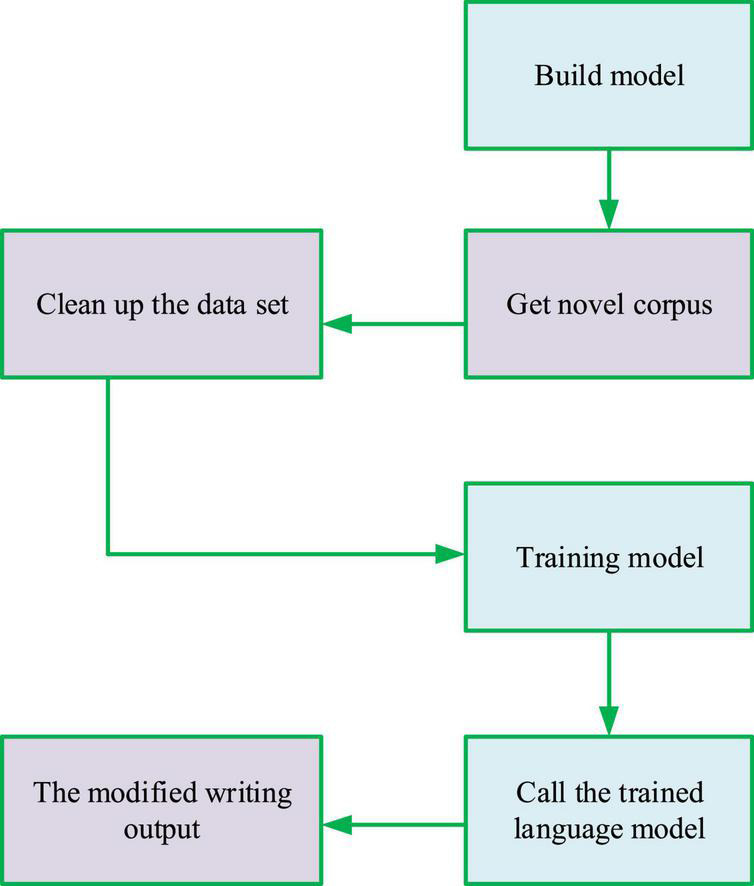
Design and implementation of intelligent modification system.

The design and implementation of the overall intelligent modification model system are drawn in Figure 8.

The overall process is shown in Figure 8, including the creation of the model, AAL TN literary corpus, data cleaning, model training, model calls, and output of modified text. This study uses the model training data set of 10,000 TN novels in AAL literature.

This study adopts the DL algorithm in AI technology; much often, DL algorithms are very time-consuming; thus, they have a higher requirement for hardware and software configurations. Accordingly, the experimental software environment is configured as follows: Python 3.6 is selected for development language, and the development tools include Terminal, Pycham 2019.2, and MobaXterm; the development framework adopts the DL Pytoch 0.4 framework; the Ubuntu 16.04 Linux system is selected with a system kernel version 4.15. The experimental hardware configuration is manifested in Table 1.

**TABLE 1:** Server resource configuration.

Hardware name	Configuration
Server	One
CPU	Intel (R) Xeon (R) CPU E5-2620 v4 @2.10 GHz
Operating System (OS)	Ubuntu 16.04
Memory	32G
GPU	NVIDIA TITAN Xp

This section defines a clean_cn class in the system to clean up the data and realize the scalability of the system in the later stage. For the implementation of data FE, it is necessary to add references to NumPy, collections, os, and sys in the program. Specifically, NumPy mainly supports some mathematical operations; the collections module implements some specific data types to replace the built-in data types, such as dict, list, set, and tuple in Python.

#### Iterative Training of Novel Corpus

The information of model iterative training is saved in the checkpoint file (checkpoint). A checkpoint is a file generated after iterative training of the model; it saves the information after iterative training of the model; it contains three partial files: meta, data, and index. In the model training of cyclic iteration, the values of all current trainable variables should be written into the checkpoint regularly, and the checkpoint should be saved, that is, the model file should be saved. After a short period, the model parameters should be overloaded to continue the cyclic training of the novel-writing model, that is, to restore the model file. Then, the Saver class provided by TensorFlow is used to save the checkpoint and write and restore the parameter information in the training model, so that the model can continue training.

Next, the proposed model is evaluated by the cross-entropy method. Here, the tf.nn.softmax_cross_entropy_with_logits() provided by TensorFlow is used to find its cross-entropy; the output of this function is not a number but the loss of each sample in a batch. The experimental process is based on the RNN LM, and the fragment in *The Sun Also Rises* is used as the main experimental object for KE and modification.

### The Questionnaire Survey Design

Based on existing research and contents in previous sections (Chen and Deng, 2021; Chi and Zhang, 2021; Milne, 2021), this section formulates several hypotheses, as evidenced in Table 2.

**TABLE 2:** Hypothetical content.

Hypothesis number	Content
H1	Reading AAL literature plays a positive role in people.
H2	Reading AAL literature can improve English proficiency.
H3	AI modification can improve the reading sense of AAL literature.
H4	ID psychology intervention can improve the reading sense of AAL literature.
H5	The combination of AI and ID psychology can improve the reading sense of AAL literature.

Table 2 demonstrates the proposed five hypotheses, which is confirmed as follows.

This section designs a QS by reviewing related works. To name of few, a survey report designed by Chen (2019), which is on cross-cultural competence and work adaptability, is referred to Chen (2019) and is more accurate and can give specific suggestions based on the results. In contrast, a survey report by Wu and Song (2019) has investigated the four major factors affecting the use and satisfaction of social media in entrepreneurship courses (Wu and Song, 2019), which is relatively rich and reliable because of the multi-attribute of the research. Additionally, the survey report on the impact of narcissism on human personality morphology is cited (Rogoza et al., 2018), together with another investigation on the impact of narcissism on entrepreneurship (Wu et al., 2019). The above survey reports have contributed partly to the QS design and the survey results in this study.

#### Questionnaire Survey Design

The survey report form is employed here, including the following three aspects: (A) Basic information of the respondents. (B) The current situation of AAL literature. (C) Reading impressions of the selected fragments.

Part A is set as the fill-in-the-blank questions for the respondents, which is convenient to classify the readers as per age and educational background. Part B is to fill in the blanks for selections, that is, apart from options setting, blanks are added and filled in by the respondents. The survey contents include reading types, reading time, reading approaches, and reading motivation. Part C is the primary part of the QS.

Later, a short TN work is selected from the original AAL literature and modified using individual AI technology and ID psychology, as well as the combination of the two, without changing the central idea and the context of the story. Meanwhile, different page formats and different story rhythms are designed, together with a QS to study the evaluations of readers of AAL TN literature from the modern perspective.

Being the first time to conduct such a survey, the QS design may not perfect. It is hoped that the QS can be improved in future research to be more scientific, reasonable, concise, and accurate.

#### Purpose of the Survey

The purpose is to facilitate the development of TN literature, so all age groups are surveyed to collect sample data comprehensively. The survey subjects are three generations of elderly, middle-aged, and young people, who have the habit of reading (hereafter referred to as active readers), with a ratio close to 1:4:5. The survey approach has mainly adopted the online distribution supplemented by an offline small sample survey.

#### Data Processing

Overall, 200 QSs are distributed, and 189 are recovered, with a recovery rate of 94.5%. Six invalid QSs are excluded, and finally, 183 valid QSs are obtained, with an effective rate of 91.5%. Thus, the QS is feasible. Subsequently, reliability analysis is conducted (Salomon et al., 2021). The calculated reliability coefficient is 0.816. Generally, a reliability coefficient less than 0.6 is considered unacceptable. The reliability coefficient between 0.7 and 0.8 is basically acceptable. The reliability coefficient between 0.8 and 0.9 is very credible. Here, the reliability analysis (Jairo et al., 2020) proves that the designed QS has high reliability. The statistical significance test is conducted later. The sample size exceeds 30, so the *z* test is selected. The calculation of the *z* test is shown in the following equation:


(6) $$\text{Z}=\frac{\bar{{\text{X}}_{1}}-\bar{{\text{X}}_{2}}}{\sqrt{\frac{{\text{S}}_{1}^{2}}{{\text{n}}_{1}}+\frac{{\text{S}}_{2}^{2}}{{\text{n}}_{2}}}}$$

where $\bar{{\text{X}}_{1}}$ and $\bar{{\text{X}}_{2}}$ represent the average of the experimental group and the control group, respectively; ${\text{S}}_{1}^{2}$ and ${\text{S}}_{2}^{2}$ denote the standard deviation; *n*_1_ and *n*_2_ stand for the capacity. If Z > 1.96, it is statistically significant. Statistical Product and Service Solutions (SPSS) 25.0 is used for data analysis.

## Results and Analysis

### Model Performance Evaluation Results

**FIGURE 9:**
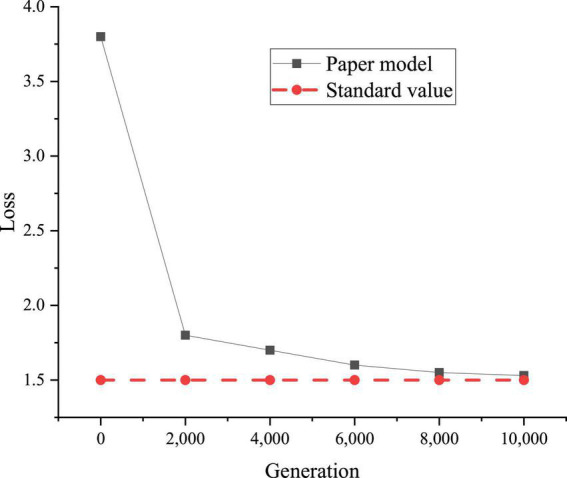
Model loss.

Figure 9 plots the loss curve of the modification LM based on the RNN.

Figure 9 reveals that the model loss of the proposed RNN-based modification LM converges to 1.5 after 10,000 iterations; the model loss is in excellent condition and can be used for subsequent modification experiments.

### Status of the Respondent

The basic information of the respondents collected is mentioned in Table 3.

**TABLE 3:** Basic information of respondents.

Classification standard		Proportion/%
Gender	Male	50.7
	Female	49.3
Age	Young people (20–35 years old)	50.4
	Middle-age people (35–55 years old)	40.5
	Elderly people (55–70 years old)	9.1
Educational background	Junior middle school	7.7
	High school (Vocational school)	16.3
	Junior college	22.4
	Bachelor	48.9
	Master and above	4.7
Monthly disposable income	Below 156.5 USD	16.7
	156.5–469.8 USD	48.9
	469.8–783 USD	21.1
	783–1566 USD	9.9
	1566–3132 USD	2.1
	Above 3132 USD	1.3

Table 3 demonstrates that under an even gender ratio, undergraduates account for the highest proportion of 48.9%, and readers with a monthly disposable income of 156.5–469.8 USD account for the most, 48.9% of overall active readers. Apparently, surveyed active readers are characterized by young and middle-aged groups, with a bachelor’s degree and a monthly salary of 156.5–469.8 USD. They mainly read AAL literature TN over the Internet.

### The Questionnaire Survey

The QS on the current situation of AAL literature is divided into two parts. Part one is the current situation of the AAL literature reading, as reflected in Tables 4, 5, which represent the reading time and types, and the current reading situation.

**TABLE 4:** Statistics of literature reading time and types.

Questions	Options	Proportion/%
(1) Average reading time per day	Less than an hour	42.7
	One to two hours	50.4
	Two to three hours	4.3
	More than three hours	2.6
(2) Reading categories	Literature	38.9
	Entertainment	31.2
	Professional technology	22.4
	Popular science	7.5
(3) Reading genres	Chinese works	68.7
	AAL literature	9.5
	Other	21.8

**TABLE 5:** Current reading situation of AAL literature.

Questions	Options	Proportion/%
(1) What is your reading impression of AAL literature?	Not practical, never read.	15.8
	It is hard to understand and seldom read.	23.1
	Have some understanding and read some.	28.7
	Very interested and frequently read.	32.4
(2) What improvements have been gained from reading AAL literature?	Become more interested in reading.	21.9
	Reading becomes better.	20.8
	The reading range becomes wider.	32.7
	The purpose of reading becomes more accurate.	24.6
(3) What is the influence of reading AAL literature on individuals?	Have changed a lot.	30.3
	Have changed slightly.	54.7
	Nothing has changed.	13.2
	There are some disadvantages.	1.8

Tables 4, 5 corroborate that as a reading habit, the literature reading is the mainstream; but the number of AAL literature readers is limited to just over 30% of the respondents, and much shorter reading time has been allocated on AAL literature than Chinese literature. The main purpose of reading AAL literature is to broaden their horizon, through which their interest and proficiency in reading are improved substantially. Yet, the motivation for reading AAL literature is low. However, AAL literature can have a positive impact on the psychology of readers, and nearly 85% of the respondents think that by reading AAL literature, they have experienced favorable changes more or less. Thus, hypothesis 1 holds. This is consistent with the research results of Augustinus (2020) that reading has a positive impact on the psychological wellbeing of people.

Next, the survey of the expectations and problems in reading AAL literature is depicted in Table 6.

**TABLE 6:** The expectations and problems in reading AAL literature.

Questions	Options	Proportion/%
(1) Is reading AAL literature helpful to your English learning?	Helpful	96.9
	Not helpful	0
	Uncertain	3.1
(2) What do you want from the AAL literature reading? (Multiple choice)	Understand the history of AAL literature	61.2
	Learn about the AAL cultural background	88.3
	Improve your English	60.5
	Improve humanistic quality	74.8
	Other	6.7
(3) What do you think are the problems or learning difficulties in reading AAL literature? (Multiple choice)	The language barrier and difficult to understand	28.4
	Lack of cultural background and difficulty to understand.	75.6
	The literature phenomenon is complex, and the context is not clear	70.1
	Don’t understand literature knowledge and cannot appreciate it	56.8

Table 6 implies that reading AAL literature can improve English reading proficiency; many readers lack an understanding of AAL literature itself and the cultural and historical knowledge behind it (Wei, 2021). Although most readers hope to understand the culture and history behind AAL literature and improve their comprehensive quality, due to the complex literary phenomenon and lack of cultural background and literary knowledge, they have chosen to give up. Hence, hypothesis 2 holds.

### Reading Impressions

**FIGURE 10:**
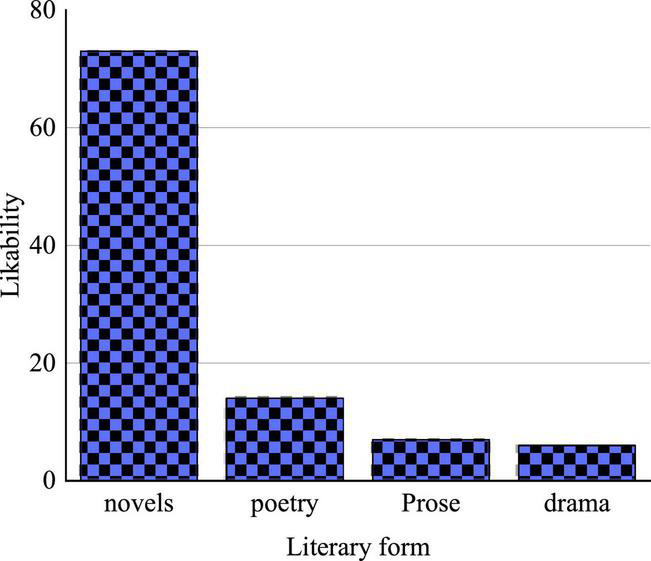
A statistical chart of the results of the pre-survey on the popularity of different forms of AAL literature.

Before the formal QS, a pre-survey is conducted on the preference AAL literature forms of respondents, as illustrated in Figure 10.

Figure 10 indicates that novels are loved by more than 70% of the respondents, and poetry is preferred by more than 10%, while prose and drama are chosen by less than 10%. Apparently, AAL novels are the favorite of respondents in terms of literature form (Larissa et al., 2020).

**FIGURE 11:**
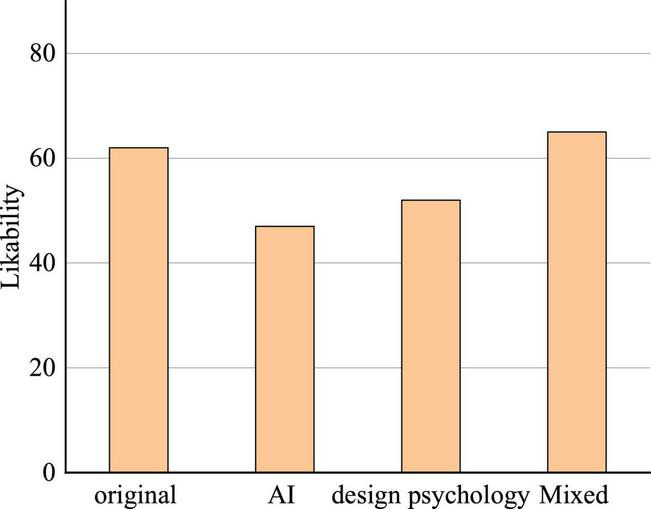
Statistical chart of likability score survey after modification by different approaches.

Consequently, the highly appraised novel *The Sun Also Rise* by Hemingway, a representative of TN literature, is specifically chosen as the original experimental work. Later, three modification methods, namely, AI automatic repair, ID psychology layout, and synergic modification by AI and ID psychology, are carried out on the premise of retaining the original meaning of the article while modifying its layout and structure. Finally, the likability scores are collected, and the advantages and disadvantages of the three revisions are summarized. Figure 11 calculates the likability scores.

Based on Eq. (1), the *z* in Table 7 is 2.16 > 1.96, so Figure 11 has a statistical significance. Figure 11 illustrates that the original version without modification gets 62% likability, which proves that the selected novel is indeed loved by most people. Comparatively, the AI-modified and ID psychology-modified versions get 47 and 53% likability, respectively, which are lower than that of the original, while the synergic-modification version reaches up to 65% likability, slightly higher than that of the original.

**TABLE 7:** Hypothesis test results.

Hypothesis number	Content	True or false
H1		True
H2	Reading AAL literature can improve English proficiency.	True
H3	AI modification can improve the reading sense of AAL literature.	False
H4	ID psychology intervention can improve the reading sense of AAL literature.	False
H5	The synergic modification of AI and ID psychology can improve the reading sense of AAL literature.	True

According to the QS results, most respondents think that the AI-modified version has moderated the overall plot and streamlined the details (Constantine, 2019). Although the reading threshold is lowered, the revision may appear imprecise. Meanwhile, the identical and mechanical text description fails to shape characters as vivid as the original, and the modification version seems like an unfinished work pieced up by the words of other literature. Worse still, there are even abrupt changes in the style of writing and unsmooth narration in some segments. Thus, hypothesis 3 is not tenable.

Interactive design psychology modifies mostly the page structure and the rhythm of the selected fragments. Compared with the original version, the revision looks briefer, tidier, and better overall impression, so the likability score has increased slightly compared with the AI-modified version (Yang et al., 2021). Nevertheless, the revision is a far cry from the original text. The feedbacks of respondents are summarized as follows: the ID psychology-based modification version is more concise and easier to read, and the main storyline is extremely clear so that readers are more likely to remember the main storyline; however, the details might have been over pruned, and no emotions as strong as the original text can be felt to produce associations, resonation, and aftertaste. Thus, hypothesis 4 is not tenable.

Finally, after the synergic modification of AI and ID psychology, the likability score is finally higher than that of the original slightly. Most readers think that after AI embellishment and ID psychology typesetting (Thomas, 2019), the focus of the mainline is more prominent, while the description of details is also improved. Compared with the original version, this revision may be improved but only less obviously. Hence, hypothesis 5 holds.

Based on the results and discussion, the verification results of the five hypotheses are given in Table 7.

Table 7 suggests that among the five proposed hypotheses, hypothesis 3 and hypothesis 4 are not tenable, while hypothesis 1, hypothesis 2, and hypothesis 5 are tenable. That is, reading AAL literature plays a positive role in the individual while improving their English proficiency concurrently; the literary modification based on single AI technology or ID psychology cannot improve the reading sense of AAL literature, but the literary modification based on the synergic efforts of the two techniques can slightly improve the reading sense of AAL literature. The research conclusion is consistent with that of Joseph (2020) and Justin et al. (2020): the reading of AAL TN literature can improve the English proficiency of people, and AI cannot completely replace humans in writing.

## Discussion

As a relatively new literary form, TN has good reasons and conditions for development; in present era of continuous S&T innovation, AI is making its way toward literary creation. The arrival of the era of intelligent literature (Wiafe et al., 2020) remains to be discussed. The study of AAL TN literature based on AI provides new ideas and influence for literary development.

Through the synergic modification of AI technology and ID psychology, this study explores the future development orientation of TN in AAL literature. The improved RNN structure is used to solve the long-time dependence of the model in the training process and the problem of too much weight data. The proposed LM employs the improved double-layered RNN structure to replace the original one-layer network structure and reduces the weight calculation of the model by increasing the depth of LSTM. Consequently, the proposed modification LM based on RNN has presented excellent performance. Thereupon, an intelligent modification system is designed and implemented.

Statistics show that in China, foreign literature, such as AAL TN literature, has a narrow audience. Surprisingly, most of them come from low-income social groups, including some undergraduates. Then, the results of the QS are summarized as follows.

The results show that reading TN novels in AAL literature has a positive impact on readers; most readers choose AAL TN literature to broaden the scope of reading and clarify the purpose of reading; few readers choose AAL literature out of their self-interests or to improve their reading skills. The main reason is the lack of literary knowledge and cultural background to appreciate the aesthetics of AAL TN literature, as well as the language barrier. For example, due to language barriers, nearly 30% of respondents plan to reduce or give up reading AAL literature.

Arguably, the most intuitive benefit of reading TN AAL literature is the improvement of English proficiency, which is not yet the main motivation. More and more people hope to gain a comprehensive and realistic understanding of the background and knowledge of AAL TN literature or improve their humanistic quality.

The main part of the QS is about the modification effect of AI and ID psychology on AAL TN literature. The results indicate that due to the limitations of current technology and ML algorithms, the literary works modified by AI cannot retain the original literary meaning: although the overall meaning of the work has not been changed by AI, many literary characters become more or less identical, thereby losing their humanistic value. In other words, the modification work is more like a copy of the same template, so it lacks much literary value.

In contrast, the overall impression of the modified version by the ID psychology is better, and the story context is clearer; however, the modified version is similar to a tree with a bare trunk, with most of its branches and leaves trimmed. At the same time, the ID psychology-modified version further highlights the mainline and enhances comprehensibility; yet, it sacrifices the core literary value rendered by some detailed description.

Finally, the synergic modification of AI technology and ID psychology has achieved satisfactory results; this combination helps to use AI technology to polish the article while typesetting based on ID psychology, and the final likability score is slightly higher than that of the original text. The final results partially confirm the five hypotheses: reading AAL TN works plays a positive role in improving the English proficiency of people; under the synergistic action of AI and ID psychology, the reading sense of AAL TN works has improved, but the reading sense under the separate action of AI technology and psychology has not improved.

## Conclusion

Based on ID psychology, this article studies the TN in AAL literature modified by AI. Specifically, it uses the improved double-layered RNN structure to replace the original one-layer network structure, increases the depth of LSTM to reduce the weight calculation of the model, and obtains the intelligent modification LM. Thereupon, the novel modification experiment is conducted for TN in AAL literature. This article studies the feasibility and possibility of AI replacing human writers in the literary creation of AAL TN, provides a direction for the improvement of AI-assisted AAL TN literature creation, and thus, promotes the development of AI in the field of literature. The results show that the proposed modification LM has excellent performance. At the current S&T level, AI and ID psychology can indeed provide users with fresh reading experiences for AAL TN literature, such as reducing the reading threshold by simplifying the content and structure. However, the improvement effect is not obvious, which proves that AI is more suitable for assisting writing than replacing human creation. After all, the human mindset is the source of literary creation, and machines cannot control or predict literary creation. The innovation of this study is to combine AI technology with ID psychology and explore the synergy between them in the creation of AAL TN literature. However, there are some limitations. The constructed intelligent modification system lacks an effective evaluation mechanism, and the discussion on the organic combination of AI technology and ID psychology is not deep enough; there are few comparative studies on the development of AI technology and the latest technology in the current writing field. In the follow-up research, this article will study the automatic evaluation mechanism, deeply explore the field of AI writing, further analyze the combination of ID psychology and AI-assisted writing, supplement the comparative analysis of the latest research technology, and focus on the role of the integration of AI-assisted writing and related psychology in the AAL TN literature.

## Data Availability Statement

The raw data supporting the conclusions of this article will be made available by the authors, without undue reservation.

## Ethics Statement

The studies involving human participants were reviewed and approved by the Ethics Committee of Zhoukou Normal University. The patients/participants provided their written informed consent to participate in this study. Written informed consent was obtained from the individual(s) for the publication of any potentially identifiable images or data included in this article.

## Author Contributions

All authors listed have made a substantial, direct, and intellectual contribution to the work, and approved it for publication.

## Conflict of Interest

The authors declare that the research was conducted in the absence of any commercial or financial relationships that could be construed as a potential conflict of interest.

## Publisher’s Note

All claims expressed in this article are solely those of the authors and do not necessarily represent those of their affiliated organizations, or those of the publisher, the editors and the reviewers. Any product that may be evaluated in this article, or claim that may be made by its manufacturer, is not guaranteed or endorsed by the publisher.
