# Supplementary material for: Interactive Design Psychology and Artificial Intelligence-Based Innovative Exploration of Anglo-American Traumatic Narrative Literature
Source: Front Psychol. 2022 Feb 10;12:755039. doi: 10.3389/fpsyg.2021.755039 (PMC8866447; doi:10.3389/fpsyg.2021.755039)
Supplement: Supplementary file 1 [file Data_Sheet_1.docx]

**Appendix 1: QS**

**QS on Interactive Design Psychology and Artificial Intelligence-based Innovative Exploration of Anglo-American Traumatic Narrative Literature**

Dear readers:

How are you! This QS is carried out toward the goal of fully understanding the impact of artificial intelligence technology and interactive design psychology on Anglo American traumatic narrative literature, as well as summarizing and analyzing the new development direction of Anglo American traumatic narrative literature in the era of artificial intelligence. Your answer is of great importance to us. We hope you can fill in this QS according to your actual situation. We promise to keep your information confidential. Thank you!

Part A: Respondents’ basic information

1. What is your gender? ____
2. What is your date of birth? ____Month____Year
3. What is your academic degree? ____
4. What is your monthly disposable income? ____

Part B: Survey on the status quo of Anglo-American literature (Multiple choice, please tick the box)

1. What is your daily reading duration?

Less than an hour; One to two hours; Two or three hours; More than three hours

1. What type of reading do you choose?

Literature; Entertainment; Professional technology; Popular science

1. What is your reading genre?

Chinese literature; Anglo American literature; other

1. What is your impression of Anglo-American literature?

Has no practical use, and I never read it; It is difficult to understand, and I rarely read it; I have a certain understanding of it and read some; I am very interested and often read it.

1. What progress do you think you have made after reading Anglo-American literature?

Become more interested in reading; Reading becomes better; The reading range is widened; The purpose of reading becomes more accurate

1. Do you think reading Anglo-American literature has a great impact on individuals?

Great changes have taken place; Little change; Nothing has changed; Have an adverse effect

Part C: The reading impression on the selected fragments (Multiple-choice questions, please check the options)

1. Do you think reading Anglo-American literature is helpful to your English learning?

Helpful; Not helpful; Uncertain

1. What do you want from Anglo-American literature? (Multiple choices)

Understand the history of Anglo-American literature; Understand Anglo American cultural background; Improve my English; Improve humanistic quality; Other

1. What do you think are the problems or learning difficulties in reading Anglo-American literature? (Multiple choices)

Language barrier, and it is difficult to understand; Lack of cultural background, and it is difficult to understand; The literary phenomenon is complex, and the context is unclear; Do not understand literary knowledge and cannot appreciate

**Pre survey**

What kind of Anglo-American literature do you like? (Multiple choice questions, please tick the option)

Novel; Poetry; Prose; Drama

This experiment has selected the fragments in Hemingway's *The Sun Also Rises*. Please fill in the form at the end of the QS after reading.

The original version reads:

We stuffed lunch and two bottles of wine into a canvas bag and bill carried it on his shoulder. I carry a fishing rod bag and hang a net behind my back. We officially set out on the road. After passing a piece of grass, we found a path through the fields to the woods on the first hillside. We followed the small sand road across the field. The fields are undulating and covered with grass. Because the sheep are grazing here, the grass is not high. The cattle graze on the mountain. We could hear the cow's bell from the woods.

The path crosses a stream by a single wooden bridge. The original surface was planed flat, and a small tree was bent and stretched out from the opposite side to serve as a handrail. There is a shallow pond next to the stream. Tadpoles swim around at the bottom of the sand. We walked up the steep bank of the stream and through the rolling fields. Looking back, you can see the White House and the red roof of burgot. A truck passes on the white road, and the dust is flying.

After crossing the field, we passed a stream with more rapids. A sand road starts from the shoal and leads to the forest. The path we took passed another single wooden bridge downstream of the shoal, then joined the sand road, and we entered the woods.

This is a beech forest, which is very old. The ground is intertwined, and the branches on the trees are twisted. We walked along the path sandwiched by the thick trunk of the old beech. The sun shone through the branches and leaves, leaving spots on the grass. The trees are tall and luxuriant, but they don't feel dark. There are no bushes under the big trees, only flat grass, green and incomparably fresh. The towering gray trees are well spaced, like a park.

The AI-modified version reads:

We stuffed lunch and two bottles of wine into a canvas bag and bill carried it on his shoulder. I carry a fishing rod bag. After passing a meadow, we found a path through the field to the woods on the first hillside. We followed the small sand road across the field. The cattle graze on the mountain. We could hear the cow's bell from the woods.

The path crosses a stream by a single wooden bridge. The original surface was planed flat, and a small tree was bent and stretched out from the opposite side to serve as a handrail. There is a shallow pond next to the stream. Tadpoles swim around at the bottom of the sand.

After crossing the field, we passed a stream with more rapids. A sand road starts from the shoal and leads to the forest. I met the sand road and we went into the woods.

This is a beech forest, which is very old. We walked along the path sandwiched by the thick trunk of the old beech. The sun shone through the branches and leaves, leaving spots on the grass. The trees are tall and luxuriant, but they don't feel dark. There are no bushes under the big trees, only flat grass, green and incomparably fresh. The towering gray trees are well spaced, like a park.

The ID psychology-modified version reads:

We stuffed lunch and two bottles of wine into a canvas bag and bill carried it on his shoulder. I carry a fishing rod bag and hang a net behind my back. We officially set out on the road. After passing a piece of grass, we found a path through the fields to the woods on the first hillside. We followed the small sand road across the field. The fields are undulating and covered with grass. Because the sheep are grazing here, the grass is not high. The cattle graze on the mountain. We could hear the cow's bell from the woods.

After crossing the field, we passed a stream with more rapids. A sand road starts from the shoal and leads to the forest. The path we took passed another single wooden bridge downstream of the shoal, then joined the sand road, and we entered the woods.

This is a beech forest, which is very old. The ground is intertwined, and the branches on the trees are twisted. We walked along the path sandwiched by the thick trunk of the old beech. The sun shone through the branches and leaves, leaving spots on the grass. The trees are tall and luxuriant, but they don't feel dark. There are no bushes under the big trees, only flat grass, green and incomparably fresh. The towering gray trees are well spaced, like a park.

The path crosses a stream by a single wooden bridge. The original surface was planed flat, and a small tree was bent and stretched out from the opposite side to serve as a handrail. There is a shallow pond next to the stream. Tadpoles swim around at the bottom of the sand. We walked up the steep bank of the stream and through the rolling fields. Looking back, you can see the White House and the red roof of burgot. A truck passes on the white road, and the dust is flying.

The synergic modification version by AI and ID psychology reads:

We stuffed lunch and two bottles of wine into a canvas bag and bill carried it on his shoulder. I carry a fishing rod bag and hang a net behind my back. We officially set out on the road. After passing a piece of grass, we found a path through the fields to the woods on the first hillside. We followed the small sand road across the field. The fields were covered with grass, which was not high because the sheep were grazing here.

The path crosses a stream by a single wooden bridge. There is a shallow pond next to the stream. Tadpoles swim around at the bottom of the sand. We walked up the steep bank of the stream and through the rolling fields. Looking back, you can see the White House and the red roof of burgot. A truck passes on the white road, and the dust is flying.

After crossing the field, we passed a stream with more rapids. A sand road starts from the shoal and leads to the forest. The path we took passed another single wooden bridge downstream of the shoal, then joined the sand road, and we entered the woods.

This is a beech forest, which is very old. The ground is intertwined, and the branches on the trees are twisted. We walked along the path sandwiched by the thick trunk of the old beech. The sun shone through the branches and leaves, leaving spots on the grass. There are no bushes under the big trees, only flat grass, green and incomparably fresh. The towering gray trees are well spaced, like a park.

Thank you for reading, please choose your favorite versions (Please tick the type you like and fill in the advantages and disadvantages you think there are):

|  | Do you like it? | What do you think the advantages and disadvantages are? |
| --- | --- | --- |
| Original version |  |  |
| AI-modified version |  |  |
| ID psychology modified version |  |  |
| Synergic modification version by AI and ID psychology |  |  |

Thank you very much for your patience and cooperation, and wish you a happy life!
